# Developmental Changes in Electrophysiological Properties and a Transition from Electrical to Chemical Coupling between Excitatory Layer 4 Neurons in the Rat Barrel Cortex

**DOI:** 10.3389/fncir.2016.00001

**Published:** 2016-01-21

**Authors:** Fliza Valiullina, Dinara Akhmetshina, Azat Nasretdinov, Marat Mukhtarov, Guzel Valeeva, Roustem Khazipov, Andrei Rozov

**Affiliations:** ^1^Laboratory of Neurobiology, Institute of Fundamental Medicine and Biology, Kazan Federal UniversityKazan, Russia; ^2^Institut de Neurobiologie de la Méditerranée, Institut National de la Santé et de la Recherche Médicale UMR901Marseille, France; ^3^Aix-Marseille UniversityMarseille, France; ^4^Department of Physiology and Pathophysiology, University of HeidelbergHeidelberg, Germany

**Keywords:** development, excitation, barrel cortex, critical period, gap junctions

## Abstract

During development, sensory systems switch from an immature to an adult mode of function along with the emergence of the active cortical states. Here, we used patch-clamp recordings from neocortical slices *in vitro* to characterize the developmental changes in the basic electrophysiological properties of excitatory L4 neurons and their connectivity before and after the developmental switch, which occurs in the rat barrel cortex *in vivo* at postnatal day P8. Prior to the switch, L4 neurons had higher resting membrane potentials, higher input resistance, lower membrane capacity, as well as action potentials (APs) with smaller amplitudes, longer durations and higher AP thresholds compared to the neurons after the switch. A sustained firing pattern also emerged around the switch. Dual patch-clamp recordings from L4 neurons revealed that recurrent connections between L4 excitatory cells do not exist before and develop rapidly across the switch. In contrast, electrical coupling between these neurons waned around the switch. We suggest that maturation of electrophysiological features, particularly acquisition of a sustained firing pattern, and a transition from the immature electrical to mature chemical synaptic coupling between excitatory L4 neurons, contributes to the developmental switch in the cortical mode of function.

## Introduction

Development of the central nervous system is characterized by critical changes in the operational mode of cortical networks. During the second half of gestation in humans, and the early postnatal period in rodents, cortical activity shows discontinuous temporal organization (known as *tracé discontinu* in human literature), characterized by alternation between long, lasting for up to tens of seconds of quiescent states alternating with bursts of spindle or gamma oscillation synchronizing almost all neuronal firing and synaptic currents (Khazipov and Luhmann, 2006; Andre et al., 2010; Blankenship and Feller, 2010; Colonnese and Khazipov, 2012; Khazipov et al., 2013). In the somatosensory cortex, these intermittent activity bursts are primarily driven by thalamic inputs, conveying sensory feedback arising from spontaneous movements of the body and whiskers to the cortex (Khazipov et al., 2004; Milh et al., 2007; Tiriac et al., 2012). These bursts of oscillatory activity can be also evoked by external sensory stimuli (Khazipov et al., 2004; Milh et al., 2007; Minlebaev et al., 2007, 2009, 2011; Yang et al., 2009, 2013). The early cortical activity patterns support plasticity in the developing networks and are thought to participate in the activity-dependent formation of the thalamocortical networks (Minlebaev et al., 2011; An et al., 2012). Early thalamocortical activity and activation of NMDA receptors have been shown to play instructing roles in various aspects of cortical development including formation of barrels and differentiation and development of L4 neurons (Espinosa et al., 2009; Minlebaev et al., 2009; Narboux-Neme et al., 2012; Li et al., 2013; Mizuno et al., 2014; Pouchelon et al., 2014). By the end of the first postnatal week in rodents and shortly before birth in humans, the bursting pattern rapidly switches to the adult-like modus operandi of cortical activity, which is manifested by a transformation in the temporal organization of the cortical activity from *tracé discontinu* to *tracé continu*, emergence of an active cortical state, and a transition of sensory evoked responses from bursting to acuity (Colonnese et al., 2010; Minlebaev et al., 2011). Several developmentally regulated factors have been suggested which may underlie the switch in cortical modes of function, including development of arousal systems and formation of recurrent excitatory cortical circuits (Colonnese et al., 2010). However, the cellular and synaptic mechanisms involved in the developmental switch at the cortical level remain poorly understood.

Layer 4 (L4) is the main input layer of the sensory cortex, which receives and pre-processes information from the thalamus and conveys it to other cortical layers (Petersen, 2007; Feldmeyer, 2012; Feldmeyer et al., 2013). Spiny and pyramidal cells are the principal excitatory neurons in L4, which receive thalamic input and process it through interplay between their electrophysiological properties and rich excitatory recurrent connectivity under the control of inhibitory neurons. Although, L4 is the primary thalamic recipient cortical layer, thalamic projections account only for 10–20% of all excitatory inputs to L4 neurons (White and Rock, 1979; Benshalom and White, 1986), the remaining 80–90% of glutamatergic connections are established between excitatory neurons of the home barrel with a connectivity rate of about 25% (Feldmeyer et al., 1999; Petersen and Sakmann, 2000; Lefort et al., 2009). It has been suggested that reach excitatory synapses between L4 neurons are well suited for amplifying thalamic input within the home barrel and distributing it further to target cells in other cortical layers (Feldmeyer et al., 1999).

During the postnatal period, L4 excitatory neurons differentiate from the dense cortical plate by postnatal day P3 and rapidly grow during the first postnatal month (Rice et al., 1985; Espinosa et al., 2009; Mizuno et al., 2014). Thalamic inputs already invade the dense cortical plate (Erzurumlu and Jhaveri, 1990; Agmon et al., 1995), drive cortical neurons (Molnar et al., 2003), and generate characteristic bursting oscillatory cortical activity patterns (Minlebaev et al., 2011; Yang et al., 2013; Mitrukhina et al., 2014) at birth. This bursting mode of function in the L4 network persists until the end of the first postnatal week, when the developmental switch of the cortical functional mode occurs (Colonnese et al., 2010; Minlebaev et al., 2011). However, little is known about when and how excitatory L4 neurons acquire their distinct electrophysiological properties, and when recurrent connectivity between these cells emerges during development. This information is important not only for an understanding of how the local L4 circuit participates in the generation of the early activity patterns, but also for deciphering the nature of the developmental switch in the cortical mode of function.

Here, we explored the developmental changes in basic electrophysiological properties and connections between excitatory L4 neurons throughout the first two postnatal weeks focusing on the changes occurring during the transition from the immature to the adult-like mode of cortical function.

## Materials and Methods

All experimental protocols were performed in accordance with the Kazan Federal University on the use of laboratory animals (ethical approval by the Institutional Animal Care and Use Committee of Kazan State Medical University N9–2013) or by the state government of Baden-Württemberg, Germany. All efforts were made to minimize animal suffering and to reduce the number of animals used.

Thalamocortical slices 350 μm thick were prepared from P4–P15 rats as previously described (Agmon and Connors, 1991). During recordings, slices were maintained at room temperature (22–24°C). Slices were continuously superfused with an extracellular solution containing (in mM): 125 NaCl, 2.5 KCl, 25 glucose, 25 NaHCO_3_, 1.25 NaH_2_PO_4_, 2 CaCl_2_, and 1 MgCl_2_, bubbled with 95% O_2_/5% CO_2_. The pipette (intracellular) solution contained (in mM) 105 potassium gluconate, 30 KCl, 10 HEPES, 10 phosphocreatine, 4 MgATP, and 0.3 Na_2_GTP (adjusted to pH 7.3 with KOH). In all experiments performed to assess passive and active neuronal properties, pipettes were filled with solution containing (in mM): 135 K-methanesulfonate, 4 Na_2_ATP, 0.3 Na_2_GTP, 2 MgCl_2_, and 10 HEPES (adjusted to pH 7.3 with KOH).

**FIGURE 1:**
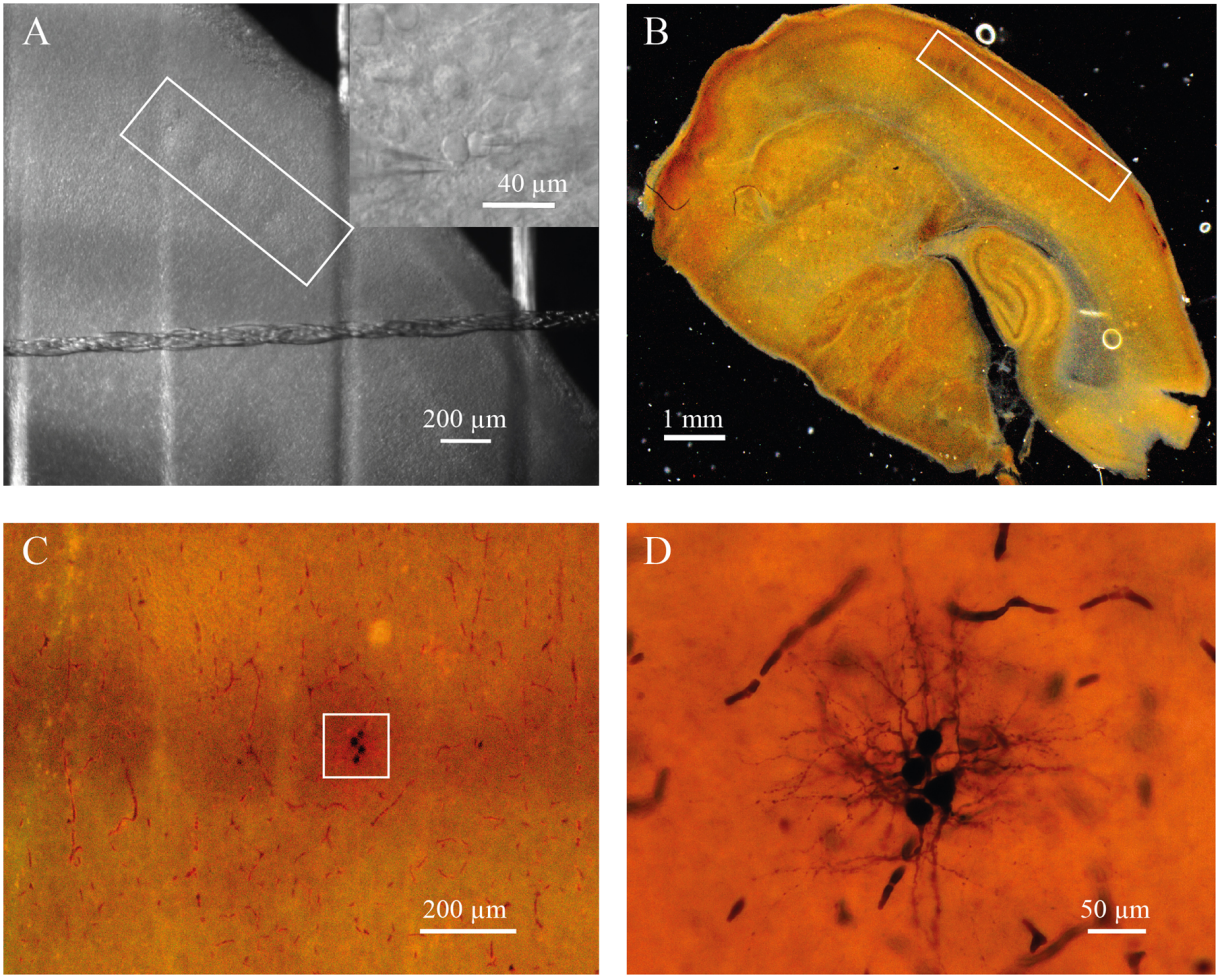
**(A)** Barrels in layer 4 of somatosensory cortex of P7 rat identified by bright-field microscopy (inside of white rectangle). Insert shows representative IR image of whole-cell recordings from the pair of identified neurons located a barrel. **(B)** Slice from P7 rat stained for cytochrome C to visualize barrels (inside of white rectangle). **(C)** The same slice as in **(B)** at higher magnification. White square indicate the barrel with four biocytin labeled cells. **(D)** The same cells as in **(C)** at high magnification.

For electrophysiological recordings, slices were placed in the recording chamber under an upright microscope (BX-51 WI; Olympus, Japan). Putative excitatory neurons were identified at 40× magnification using infrared-differential interference contrast (IR-DIC) microscopy by location, shape of cell body, and firing properties (Feldmeyer et al., 1999; Staiger et al., 2004; Daw et al., 2007). Seventy-five cells were filled with biocytin to confirm spiny stellate or pyramidal neuron morphology. Barrels were visualized by bright-field microscopy. In some slices the location of a neuron within the barrel was confirmed by cytochrome C staining (**Figure 1**; Petersen and Sakmann, 2000).

Signals were recorded using two different recording systems. The first setup was: two EPC-8 amplifiers (HEKA Elektronik, Lambrecht, Germany), filtered at 3 kHz and digitized at 10 kHz using an ITC-18 interface (InstruTECH, Mineola, NY, USA) and PatchMaster acquisition software (version 8.21; HEKA Elektronik). The second setup was: MultiClamp 700B amplifier, Digidata 1440A digitizer and pClamp 10 acquisition software (Molecular Devices, USA).

Values of the passive neuronal properties: input resistance, resting membrane potential (RMP), and membrane capacitance, were taken as they were measured by pClamp 10 acquisition software. Neurons with a RMP higher than – 40 mV were discarded from further analysis.

In paired recording experiments, pre- and postsynaptic neurons were located within 100 μm from each other. Electrical coupling was tested by injecting hyperpolarizing and depolarizing currents as previously described (Venance et al., 2000). Pre- and postsynaptic voltage responses were obtained by averaging 50 consecutive sweeps. The coupling coefficient was calculated as the ratio of the averaged spikelet amplitude in cell 2 divided by the amplitude of the action potential (AP) in cell 1.

To study synaptic connections, presynaptic cells were stimulated with a 10 Hz train of three suprathreshold current pulses every 10 s. All recordings were carried out in current clamp mode. Postsynaptic cells were kept at RMPs. Averages of 50–100 consecutive sweeps were used for the analysis of synaptic properties.

All data were analyzed oﬄine using pCLAMP (Axon Instruments, Molecular Devices, USA) or PatchMaster (HEKA Elektronik, Germany) and Origin 7.0 (Microcal Software, Northampton, MA, USA). To obtain the *P*-value map we used MATLAB R2013 (MathWorks, Natick, MA, USA). Mann–Whitney Rank Sum or Wilcoxon Rank Sum (where indicated) tests were used for statistical comparisons. The level of significance was set at *P* < 0.05. The data are presented as medians and 25th/75th percentile, unless otherwise stated.

## Results

### Developmental Changes of Intrinsic Properties and Excitability of Layer 4 Neurons

Whole-cell recordings were performed from putative excitatory layer 4 neurons in the rat barrel cortex at different developmental stages (P4–P13). Neurons were identified by location, shape of cell body, firing properties, and morphology. The excitatory neurons in layer 4 are spiny stellate cells, star pyramids, and L4 pyramidal neurons (Brecht and Sakmann, 2002; Staiger et al., 2004; Bruno and Sakmann, 2006). However, we did not observe major functional differences between excitatory L4 neurons in agreement with the results of previous studies (Feldmeyer et al., 1999; Lubke et al., 2000; Bender et al., 2003). Therefore, data obtained from all types of excitatory neurons were pooled together.

**FIGURE 2:**
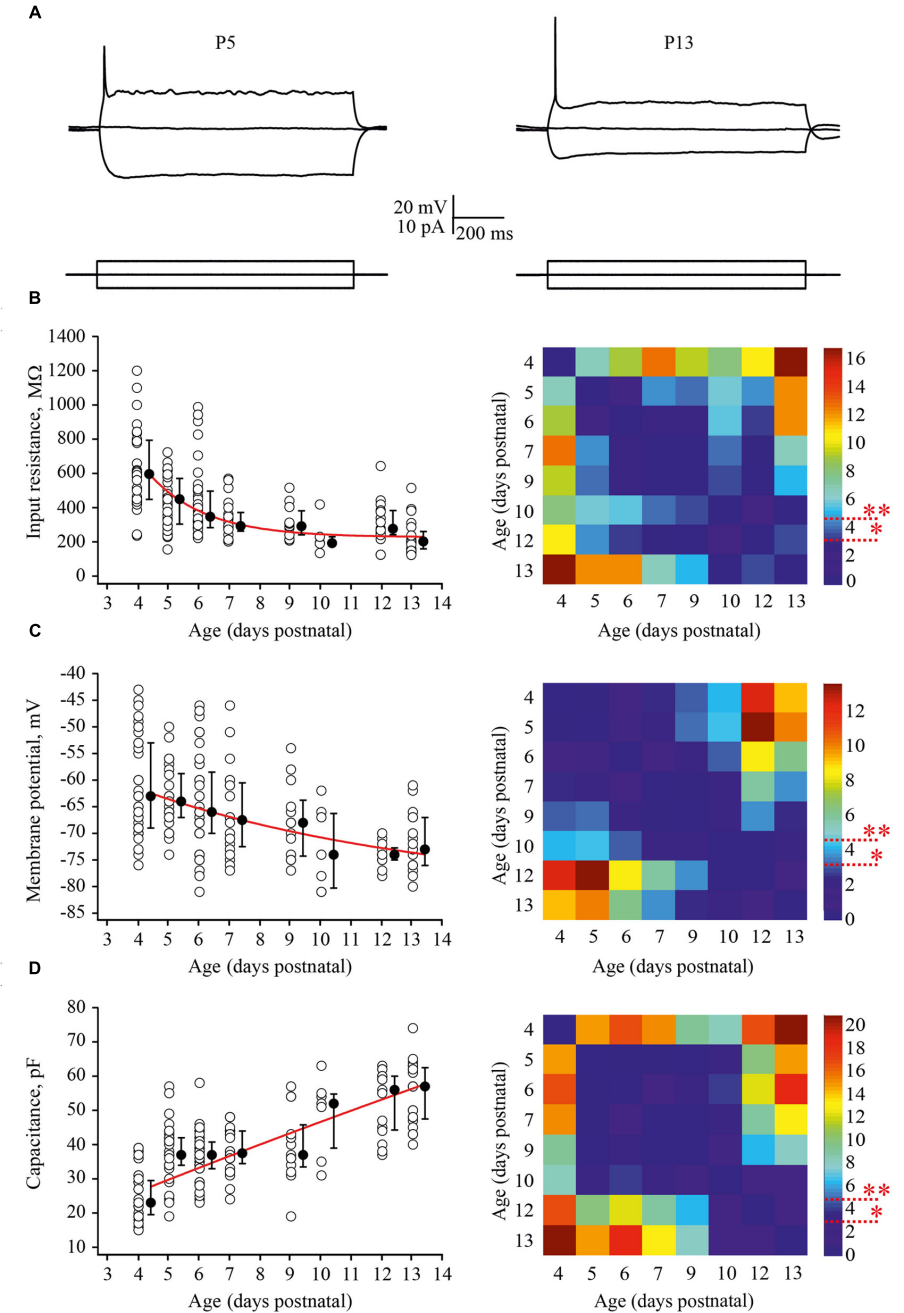
**Postnatal development of the passive L4 neuronal properties. (A)** Voltage responses (top traces) generated by 1 second current injections (bottom traces) recorded from P5 (left) and P13 (right) L4 excitatory neurons. **(B–D)** Summary graphs show age-dependent decrease of the input resistance **(B)**, decrease in the resting membrane potential (RMP) **(C)**, and an increase in the capacitance of the membrane **(D)**. Each open circle represents data from one L4 neuron. Filled circles are averages for each postnatal day. Red lines represent single exponential fits of averaged data points (left). Statistical comparisons of differences between ages. Correspondent *P*-value maps (Wilcoxon Rank Sum Test) compare age dependent differences in input resistance, membrane potential and in membrane capacitance (right). The color-coded bar represents –*lnP* values (^∗^*P* = 0.05, ^∗∗^*P* = 0.01).

Firstly, we looked at developmental changes in passive neuronal properties such as input resistance [R(in)], RMP, and membrane capacitance [C(m)] (**Figures 2A–D**). In total, measurements were obtained from 190 cells with 6–43 neurons for each age group. At P4, L4 neurons had a high averaged R(in) of 596 (477/793) MΩ [median (25th/75th percentile)] (*n* = 29). R(in) gradually decreased with each successive day of maturation reaching 202 (160/261) MΩ by P13 (*n* = 20). **Figure 2B** shows individual and averaged data points plotted as a function of age and a *P*-value map. During the first postnatal week individual values measured in P4–P7 animals (*n* = 119) were scattered over a wide range from 200 to 1200 MΩ probably reflecting vast developmental stage diversity between neurons at these ages. During the second week, the scatter of individual R(in) values was less pronounced and at P9–P13 R(in) values ranged from 100 to 600 MΩ (*n* = 52).

Before the developmental switch in the cortical mode of function occurring *in vivo* (P4–P7), L4 neurons were significantly depolarized compared with cells in more mature animals. Thus, at P4 the averaged median RMP value was –63 (–57/–73) mV (*n* = 33), while at P13 this was –73 (–70/–79) mV (*n* = 18; **Figure 2C**). Again the spread of RMP values between individual neurons was more pronounced in the younger animals. Finally, we assessed developmental changes in neuronal membrane capacitance C(m). C(m) gradually increased with age, being 23 (19.5/29.5) pF at P4 (*n* = 36) and reaching 57 (47.5/62.5) pF by P13 (*n* = 20; **Figure 2D**). Statistical analysis revealed that at age P4–P9, neurons had significantly lower C(m) when compared to the older animals (**Figure 2D**, *P*-value map).

**FIGURE 3:**
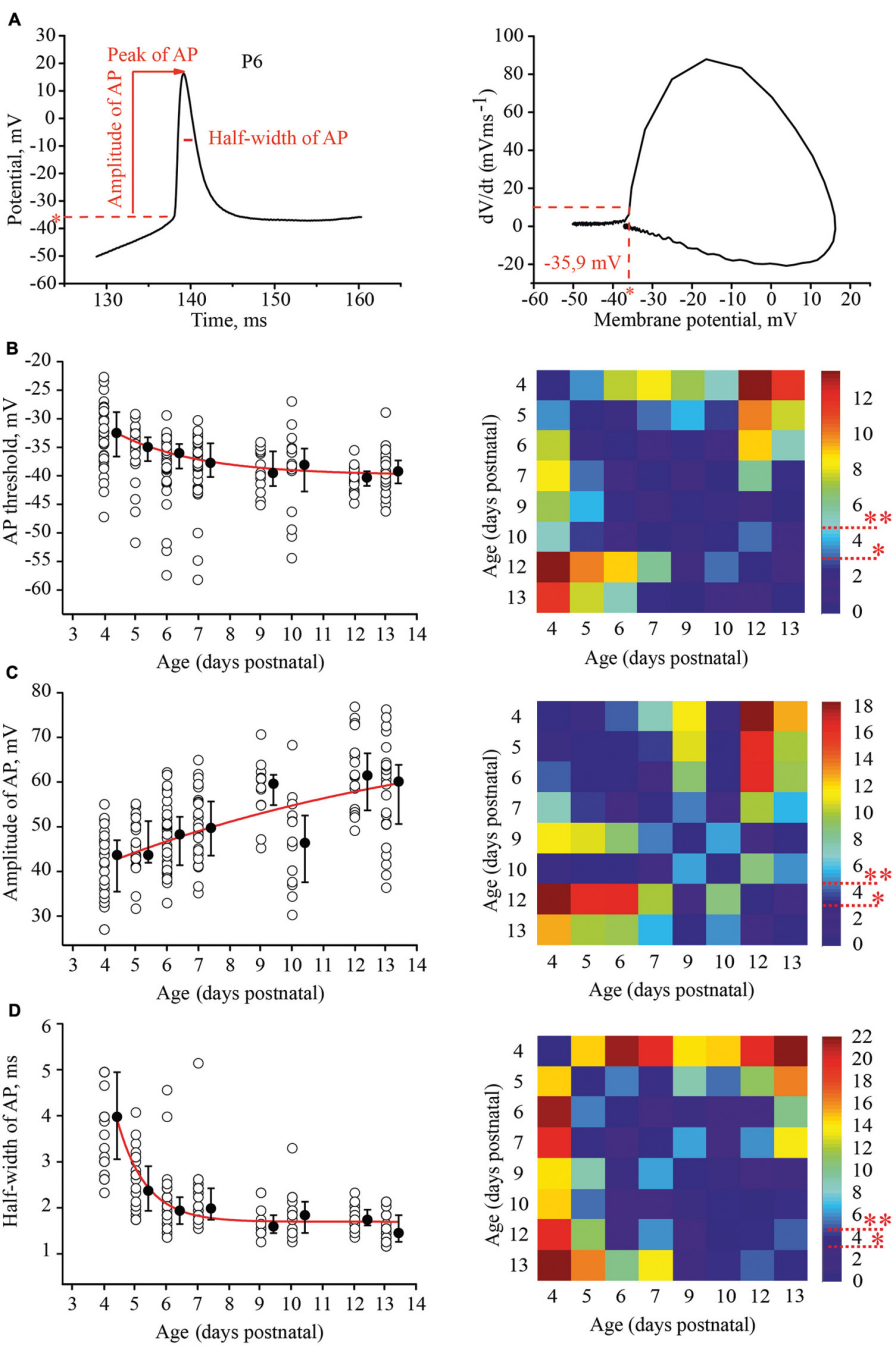
**Postnatal development of the action potential (AP) properties in L4 excitatory neurons. (A)** Example trace of the first AP evoked by the minimal suprathreshold current step. The red dash line represents AP threshold (left). AP threshold was determined by phase plot as a value of membrane potential at which dV/dt exceeded 10 V/s (Stuart et al., 1997; Fricker et al., 1999) (right). **(B–D)** Scatter plots illustrate the following AP properties as a function of age: AP threshold **(B)**, AP amplitude **(C)**, half-width of AP **(D).** Each open circle represents the value for one L4 neuron. Filled circles are averages for each postnatal day. Red lines are the single exponential fits of the averaged data points (left). Correspondent *P*-value maps (Wilcoxon Rank Sum Test) compare age dependent differences in: Threshold, amplitude and half-width of AP (right). The color-coded bar represents –*lnP* values (^∗^*P* = 0.05, ^∗∗^*P* = 0.01).

To evaluate the developmental tuning in excitability of layer 4 spiny neurons, we measured and compared the threshold of AP generation, amplitudes, and half-width of APs and firing frequencies at different ages. APs were evoked by 1 s depolarizing minimal suprathreshold current injections. For analysis we used the first AP in the train. The time of onset was defined as the time at which the rate of rise of voltage was greater than 10 V/s (**Figure 3A**). We found that the AP threshold in excitatory L4 neurons became slightly, but significantly more negative during maturation, shifting from –33.1 (–29/–36.7) mV (*n* = 33) at P4 to –39.8 (–37.7/–41.7) mV at P13 (*n* = 29; *p* < 0.01; **Figure 3B**).

Action potential amplitudes were measured from the onset to the peak. **Figure 3C** illustrates age dependence of AP amplitudes and the corresponding *P*-value map. On average, between P4 and P13, AP amplitudes increased by 16 mV from 43.7 (35.5/47) mV (*n* = 28) to 60.1 (50.6/63.8) mV (*n* = 26), respectively.

In addition to the increase in the amplitudes, AP kinetics changed during the first two postnatal weeks. They were significantly wider in younger animals. At P4 the AP half-width was 4 (3.05/5) ms (*n* = 28), while in more mature neurons at P9, the AP half-width was 1.55 (1.4/1.7) ms (*n* = 12). This value did not change significantly in older animals (at P13, 1.4 (1.2/1.6) ms; *n* = 26; *p* > 0.05; **Figure 3D**)

**FIGURE 4:**
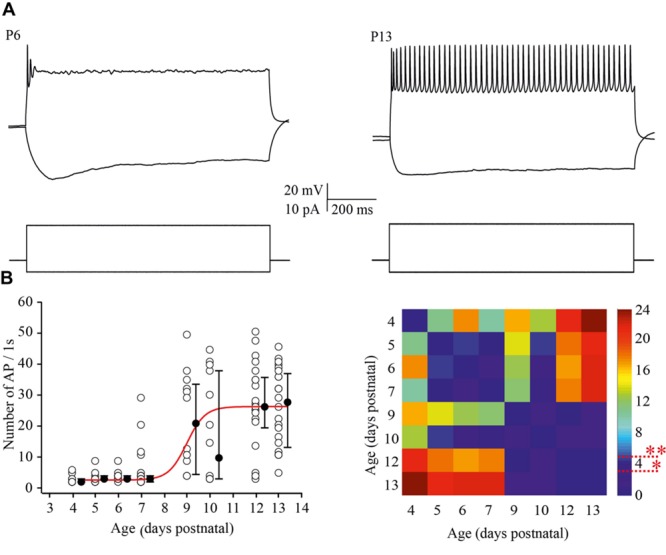
**Postnatal changes in the firing frequency of L4 excitatory cells. (A)** AP patterns recorded after a depolarizing current injection of the same value (20 pA) from P6 (left) and P13 (right) L4 excitatory neurons. **(B)** The plot shows the number of APs evoked by 1 s current injections as a function of animal age. Each open circle represents the value for one L4 neuron. Filled circles are averages for each postnatal day. Red line is Boltzmann fit of the averaged data (left). Correspondent P-value maps (Wilcoxon Rank Sum Test) compare the number of APs across tested ages (right). The color-coded bar represents –*lnP* values (^∗^*P* = 0.05, ^∗∗^*P* = 0.01).

To examine the ability of neurons to maintain firing we increased the amplitude of the depolarizing current injections by two fold relative to the minimal suprathreshold current. For analysis the number of APs that cells could fire during 1 s of steady state depolarization was measured. At P4 most of the neurons were able to fire only one, or rarely, two APs. This tendency persisted at P5–P7, although, some (25 out of 93) of the cells generated bursts of two (*n* = 22) to four (*n* = 3) APs with the first of interspike interval 11.2 ± 2.13 ms (mean ± SD). Firing ability drastically improved in >P8 animals, i.e., at the moment of the developmental switch, when most of the tested neurons maintained continues firing for the entire period of depolarization (**Figure 4A**). The data and statistical analysis are summarized in **Figure 4B**.

Thus, during the period of the immature bursting mode of function in the somatosensory cortex *in vivo*, L4 neurons display higher RMPs, higher input resistance, lower membrane capacity, APs with a smaller amplitude and longer duration, and a higher AP threshold and fail to sustain firing during prolonged depolarization compared to the neurons after the developmental switch to the adult-like mode of cortical function. These modifications in passive and active properties likely reflect growth of the dendritic trees, characterized by a sharp increase in the number of new branch points and total dendritic length between P6 and P9 (Espinosa et al., 2009; Mizuno et al., 2014) that likely contributes to a developmental decrease in membrane resistance and an increase in capacitance of L4 neurons. The developmental change in the expression of ionic channels, notably an age-dependent increase in potassium channel expression may account for the changes in the acceleration of the APs and the emergence of the sustained firing ability of L4 neurons (Moody and Bosma, 2005; Guan et al., 2011).

### Development of Synaptic Connectivity Between Excitatory Neurons in the Barrel Cortex

**FIGURE 5:**
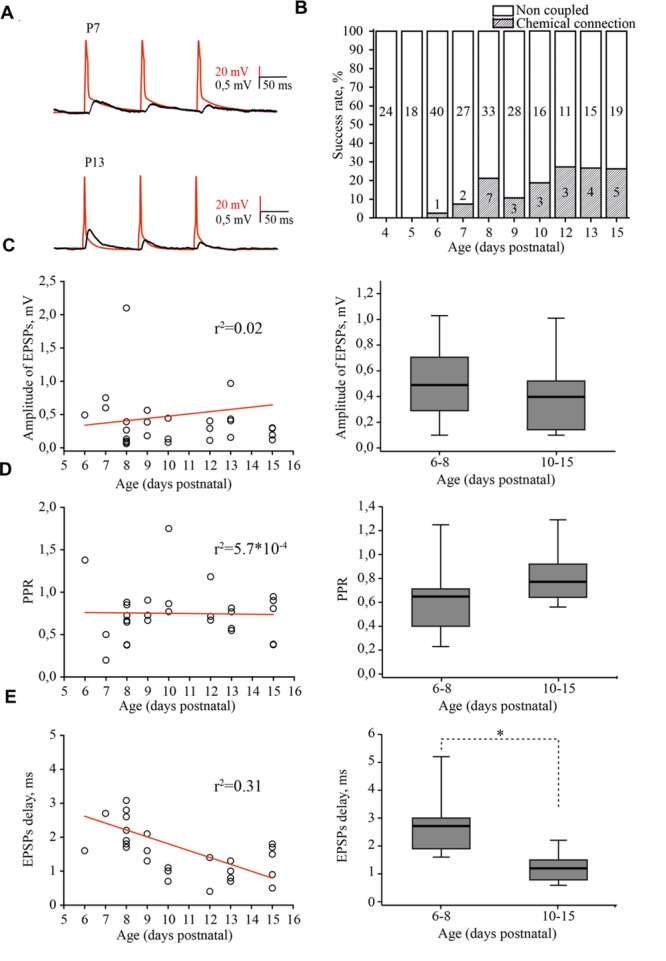
**Developmental changes in synaptic connectivity and properties of connections between excitatory neurons of the L4 rat barrel cortex. (A)** Example pairs of traces show the presynaptic APs (red) and associated EPSPs (black) recorded from the connected excitatory neurons in layer 4 at P7 (top) and P13 (bottom). **(B)** The histogram summarizes the success rate of finding chemical connections between excitatory L4 neurons at different postnatal stages. The value in the middle part of each column is the total number of tested pairs, the value in the lower part of the column indicates the number of connections found for a given animal age. **(C)** The scatter plot represents the EPSPs amplitude as a function of animal age. Each open circle represents an individual connection (left). Box plot compares averaged EPSPs amplitude for two age groups P6–P8 and P10–P15 (right). **(D)** The paired-pulse ratio (PPR) plotted as a function of animal age. Each open circle represents an individual connection (left). Box plot compares averaged PPRs for two age group P6–P8 and P10–P15 (right). **(E)** Scatter plot shows the age dependence of EPSPs delay. Each open circle represents an individual connection (left). Box plot compares averaged EPSPs delay for two age groups P6–P8 and P10–P15 (right). Red lines are a linear regression fit of the data. Box plots: the box indicates the 25th, 50th (median), and 75th percentiles, the error bars indicate the 10th and 90th percentiles. Asterisks indicate significant difference.

Next, we investigated the development of intrinsic synaptic connections between layer 4 excitatory neurons in the barrel cortex using the paired recording technique (**Figure 5A**). Presynaptic cells were stimulated by 10 Hz trains of suprathreshold depolarizing pulses. The averaged peak amplitude of the unitary excitatory postsynaptic potential (EPSP), evoked by a single presynaptic AP (the first AP in the train) was taken as an index of efficacy.

In simultaneous whole-cell paired recordings from 42 pairs, in slices from P4 and P5 animals, synaptically connected neurons were not found. The first evidence of synaptic connection between the L4 neurons was found in P6 animals, in 1 out of 40 tested pairs (2.5% connectivity). The success rate of finding connected neurons increased in an age-dependent manner, reaching the adult-like value of 25% by P12 (5 out of 19 pairs; **Figure 5B**; Feldmeyer et al., 1999; Lefort et al., 2009). EPSP amplitudes were in the range of 0.06–3.42 mV, which is comparable to reported adult values of 0.06–7.79 mV (Lefort et al., 2009) and did not depend on the age of the animals (**Figure 5C**).

Most of the connections showed paired-pulse depression, again regardless of the developmental stage (**Figure 5D**). The frequency-dependent change in efficacy was evaluated as a property of synapses that is thought to be determined mostly by a presynaptic mechanism (reviewed in Zucker and Regehr, 2002). **Figure 5D** illustrates the distribution of the paired-pulse ratios (PPRs) of EPSPs. The averaged PPR in P6–P8 animals was 0.65 (0.41/0.71; *n* = 10), while in pairs from P10–P15 rats, the averaged PPR was 0.77 (0.65/0.93; *n* = 15) similar to that previously reported for P13–P15 animals (Petersen, 2002). However, we found that synaptic delay in pairs between L4 excitatory neurons significantly shortened with maturation. In pairs recorded in slices from P6–P8 rats, the averaged EPSP delay was 2.7 (1.9/3) ms (*n* = 10), which was nearly two times longer compared to the value obtained in P10–P15 animals [1.2 (0.8/1.5) ms; *n* = 15; *p* < 0.01; **Figure 5E**].

Thus, L4 excitatory cells are poorly coupled via glutamatergic synapses during the first postnatal week and synaptic connectivity develops rapidly around the developmental switch to reach adult values by P12. Our results also indicate that, similarly to the development of thalamocortical inputs to L4 cells (Crocker-Buque et al., 2015), L4–L4 excitatory connectivity growth occurs through an increase in the number of connections without a change in the weight of individual connections.

### Electrical Coupling Between Excitatory Neurons in the Barrel Cortex at Early Postnatal Ages

**FIGURE 6:**
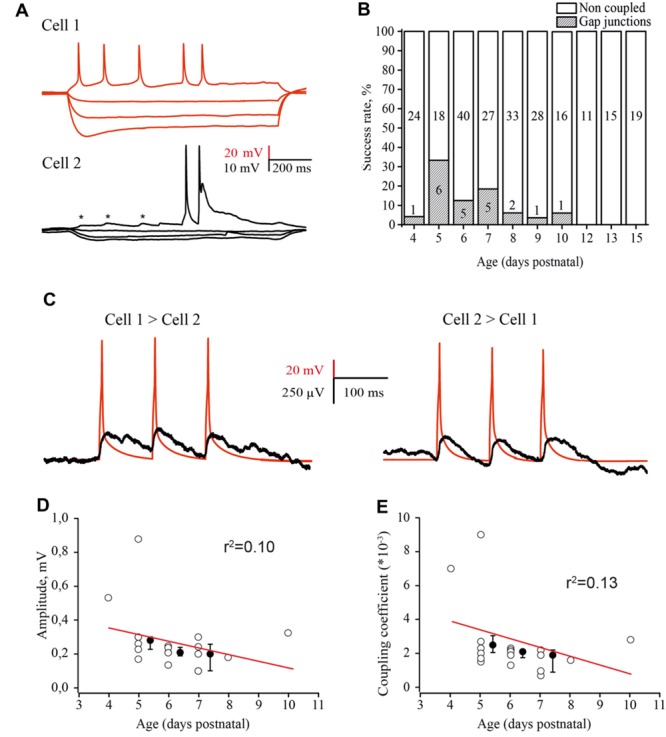
**Developmental changes in electrical coupling between L4 excitatory neurons in the rat barrel cortex. (A)** Paired recordings of an electrically coupled pair of excitatory neurons. Voltage responses to hyperpolarizing and depolarizing currents injected in the first cell (red traces) and simultaneously recorded membrane potential changes in the second cell (black traces). Asterisks indicate the spikelets emerging in response to the AP in the presynaptic neuron. **(B)** The histogram summarizes the probability of finding electrical connections between excitatory neurons in layer 4 of the cortex at different stages of postnatal development. The value in the middle part of each column is the total number of tested pairs, the value in the lower part of the column indicates the number of connections found for a given animal age. **(C)** Example traces of DC coupling between gap junction connected neurons in layer 4 of the barrel cortex in slices of P5 rat. The activation of a presynaptic neuron by three suprathreshold current pulses generated the train of APs (red trace), which evoked a train of spikelets in the postsynaptic neuron (black trace). The traces are the averages of 100 consecutive sweeps. **(D–E)** Amplitude of postjunctional spikelets **(D)** and value of the coupling coefficient via electrical synapses **(E)** as a function of animal age. Each point represents an individual connection. Filled points are averages for each postnatal day. Red lines are a linear regression fit of the data.

Although the chemical synaptic connectivity between layer 4 excitatory neurons during the first postnatal week was rather low, these cells were extensively electrically connected. Electrical coupling was characterized by DC shifts and spikelets during paired recordings (**Figure 6A**). Electrical coupling showed bell shaped developmental profile: a few (1 of 24 tested pairs, 4.2%) connections observed at P4; the peak at P5 (**Figures 6A,B**), when 33% of tested pairs were electrically coupled (6 out of 18 pairs); and a progressive decline with age so that at P9–P10, electrical coupling was detected in only 4.3% of the tested pairs (2 out of 44). At later developmental stages (P11–P15) no electrical connections between excitatory cells were observed (*n* = 35; **Figure 6B**) this is in agreement with previous studies which also reported glutamatergic but not electrical coupling between L4 excitatory neurons in animals >P12 (Feldmeyer et al., 1999; Petersen and Sakmann, 2000; Lefort et al., 2009).

The coupling coefficient was calculated as the ratio of spikelet amplitude in the postsynaptic cell to the amplitude of AP in the presynaptic neuron. The coupling coefficient was the same for all three APs in the train and comparable in both directions (**Figure 6C**). The amplitudes of spikelets and the coupling coefficient were slightly, but not significantly higher in younger animals (**Figures 6D,E**) and were comaparable with the efficacy of electrical coupling between neonatal L5 pyramidal cells (Yu et al., 2012).

## Discussion

**FIGURE 7:**
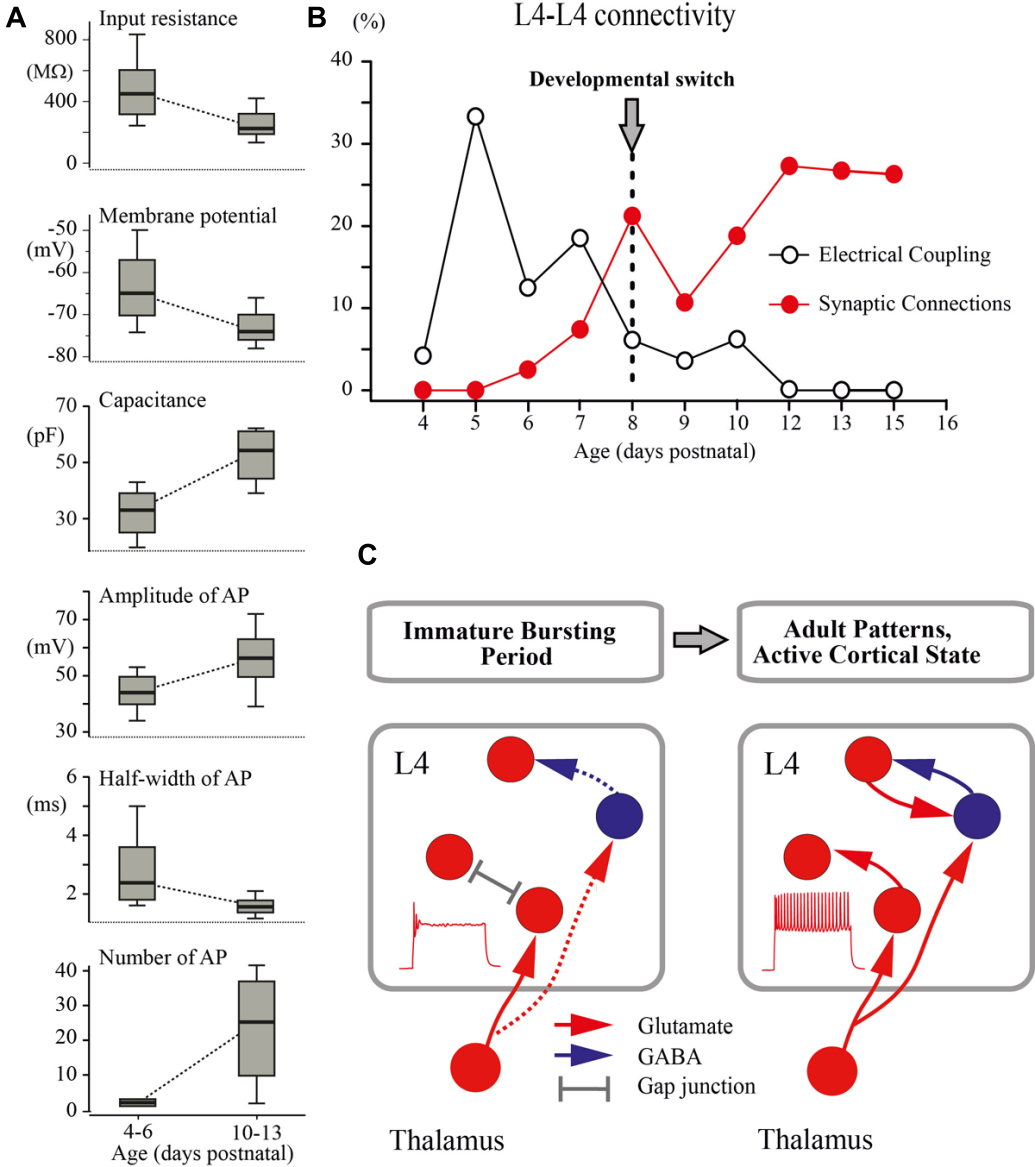
**Developmental switch in the intrinsic properties and connectivity of L4 excitatory neurons. (A)** The box plots compare averaged input resistance, RMP, capacitance of the membrane, averaged AP amplitude, half-width of AP, and number of AP for two age groups P4–P6 and P10–P13. The box indicates the 25th, 50th (median), and 75th percentiles, the error bars indicate the 10th and 90th percentiles. **(B)** Age dependence of the probability of finding glutamatergic (red circles) and electrical (gray circles) synapses between L4 excitatory neurons in slices of the barrel cortex. **(C)** Proposed changes in the L4 circuit during a switch from the immature to the adult-like mode of operation in the somatosensory system. During the immature bursting period, regular firing properties of L4 neurons and excitatory/inhibitory local connections within L4 are poorly developed. At this stage, the L4 network is primarily driven by thalamic input and operates on a reflexological basis. The developmental switch to the adult-like mode of function is associated with acquisition of the sustained firing patterns by L4 neurons and formation of local excitatory and inhibitory connections that enable the L4 circuit to generate internally organized activity patterns.

The main finding of the present study is that a developmental switch from the immature to the adult-like mode of cortical function, which occurs in the rat somatosensory cortex *in vivo* at around P8 (reviewed in Colonnese and Khazipov, 2012; Khazipov et al., 2013), is associated with a cardinal change in the L4 excitatory network involving maturation of the intrinsic electrophysiological properties of L4 neurons leading to the acquisition of a sustained firing pattern and a switch from immature electrical coupling to chemical synaptic connections between excitatory L4 neurons (**Figure 7**).

The developmental changes in the intrinsic electrophysiological properties of excitatory L4 neurons and connections between them reported here may have a strong impact on the developmental changes in the cortical network function. In particular, they may contribute, at the cortical level, to the developmental switch from immature to the adult-like mode of sensory processing and an emergence of the active cortical state described in the somatosensory cortex *in vivo*. Previous studies in the neonatal somatosensory cortex *in vivo* revealed characteristic intermittent patterns of spindle-bursts and early gamma oscillations (EGOs; Khazipov et al., 2004, 2013; Minlebaev et al., 2007, 2009; Yang et al., 2009, 2013; Colonnese and Khazipov, 2012; Gerasimova et al., 2014; Mitrukhina et al., 2014; Sitdikova et al., 2014). Whole-cell recordings from L4 neurons revealed that these activity patterns are primarily driven by glutamatergic connections (Minlebaev et al., 2011). The source of the glutamatergic inputs driving the early activity patterns in the L4 circuit remained unknown until the present study. It potentially involves thalamocortical and local L4–L4 excitatory connections, which account for 15% and 85% of all excitatory inputs to L4 neurons in adults, respectively (Benshalom and White, 1986; Feldmeyer et al., 1999; Lefort et al., 2009). While the early formation of the thalamic inputs to L4 neurons (Agmon et al., 1993, 1995; Isaac et al., 1997; Daw et al., 2007; Erzurumlu and Gaspar, 2012) and the importance of the thalamic input to L4 in the generation of EGOs and spindle-burst oscillations (Minlebaev et al., 2011; Yang et al., 2013) have been well evidenced, the role of the local excitatory L4 circuitry in supporting these network activities has remained unknown. Here, we found that during the first postnatal week, recurrent glutamatergic connectivity between L4 neurons is virtually non-existent. The first few connections were found at P6–P7, shortly before the switch. This indicates that a recurrent excitatory L4 network is poorly developed, and therefore early oscillations observed *in vivo* are mostly driven by the down–up stream of thalamic excitation. This also indicates that the first glutamatergic synapses formed on L4 neurons and described in morphological studies (Dufour et al., 2015) are of thalamic origin. These findings are also in agreement with the observations indicating the instructing roles of thalamocortical but not intracortical glutamatergic synapses (Narboux-Neme et al., 2012; Li et al., 2013; Pouchelon et al., 2014) and particularly NMDA receptors, which are activated during the intermittent bursts (Espinosa et al., 2009; Minlebaev et al., 2009; Mizuno et al., 2014) in the differentiation and development of L4 neurons during this early developmental period.

We found that L4 neurons are transiently coupled via electrical synapses during development, with a peak of connectivity attained at around P5. Similar bell-shaped developmental profile has been previously reported for the rat visual cortex, where the highest levels of the presumable gap-junction dye-coupling were observed between P5 and P12 with a steady decline at P18 (Peinado et al., 1993; Peinado, 2001), and in the rat hippocampus at around birth (Crepel et al., 2007; see also Yu et al., 2012 for L5 pyramidal cells). While the mechanisms underlying transient electrical coupling between L4 neurons during development are unknown, it may be suggested that it depends on one hand on the dendritic growth of L4 neurons (Espinosa et al., 2009; Mizuno et al., 2014) and on the other hand on transient expression of Connexin 26 (Nadarajah et al., 1997). Several correlated activity patterns, synchronized through gap junctions, have been described in slices of the developing cortex *in vitro*, including correlated calcium waves, neuronal domains, spontaneous plateau assemblies, and carbachol induced beta oscillations (Yuste et al., 1992; Kandler and Katz, 1998; Peinado, 2001; Dupont et al., 2006; Crepel et al., 2007). However, the existing experimental evidence indicates that electrical coupling plays a limited role in the generation of the early activity bursts as neither mefloquine (Minlebaev et al., 2007) nor carbenoxolone (data not shown) significantly affected early *in vivo* activity patterns. The physiological role of this transient electrical coupling between L4 excitatory neurons is at present unknown, but it may be suggested that it serves as a template for further synaptic coupling as has been previously shown for the infragranular layers, where electrical transmission between sister pyramidal cells in ontogenic columns is required for the development of precise chemical synapses between them (Yu et al., 2012).

Interestingly, inhibitory circuits are also only recruited to the L4 network by the end of the first postnatal week (Daw et al., 2007; Minlebaev et al., 2011). It has been shown that in thalamocortical slices from P3–P5 animals, thalamic stimuli evoke purely monosynaptic responses in L4 cells that are unaltered after blockade of GABA(A) receptors, whereas in older animals, at P7–P9, blockade of inhibition leads to emergence of polysynaptic epileptiform bursts (Daw et al., 2007). Similarly, in rat pups *in vivo*, blockade of cortical GABA(A) receptors did not affect EGOs and MUA-dip after the sensory-evoked potential in animals younger than P5, and induced epileptiform bursts only in animals older than P5 (Minlebaev et al., 2011). Based on our findings of delayed development in the recurrent excitatory circuitry in L4, we propose that (i) formation of local excitatory synapses provides a basis for delayed emergence of the disinhibition-induced epileptiform activities in the L4 network, and that (ii) formation of both excitatory and inhibitory local L4 circuits follow similar developmental profiles.

An important conclusion arising from these findings is that during the first postnatal week, the operation of the extremely poorly developed L4 network is fundamentally “reflexological”, as the working organization of the entire adult nervous system has been viewed early in a historical perspective by James, Sherrington and others (reviewed in Llinas, 2001; **Figure 7C**). During this early developmental period, are formed the first glutamatergic synapses on L4 neurons of thalamic origin and the activity of L4 neurons is primarily controlled by thalamic inputs, whereas the ability of the L4 network to generate internally organized activities, including continuous active states and locally generated oscillatory patterns emerges only with an acquisition by L4 neurons of the sustained neuronal firing patterns and the formation of local excitatory and inhibitory synapses at the end of the first postnatal week. We propose that the changes in cellular and local network properties occurring in L4 at the end of the first postnatal week contribute to the developmental switch to the adult-like mode of cortical function, which is primarily based on the internally generated activities with the external input playing only a modulatory role.

## Author Contributions

RK and AR: study conception and design; FV, MM, and AR: acquisition, analysis, and interpretation of the *in vitro* data; DA, AN, and GV: acquisition, analysis, and interpretation of the *in vivo* data; FV, RK, and AR: drafting of manuscript; AR: critical revision.

## Conflict of Interest Statement

The authors declare that the research was conducted in the absence of any commercial or financial relationships that could be construed as a potential conflict of interest.
